# Importance of Intermittent Fasting Regimens and Selection of Adequate Therapy on Inflammation and Oxidative Stress in SARS-CoV-2 Infection

**DOI:** 10.3390/nu14204299

**Published:** 2022-10-14

**Authors:** Armin Ezzati, Sara K. Rosenkranz, Benjamin D. Horne

**Affiliations:** 1 Department of Food, Nutrition, Dietetics and Health, Kansas State University, Manhattan, KS 66506, USA; 2 Physical Activity and Nutrition Clinical Research Consortium, College of Health and Human Sciences, Manhattan, KS 66506, USA; 3 Department of Kinesiology and Nutrition Sciences, University of Nevada Las Vegas, Las Vegas, NV 89154, USA; 4 Intermountain Medical Center Heart Institute, Salt Lake City, UT 84107, USA; 5 Division of Cardiovascular Medicine, Department of Medicine, Stanford University, Stanford, CA 94305, USA

**Keywords:** SARS-CoV-2, dietary supplements, intermittent fasting, COVID-19, inflammation, oxidative stress, time-restricted eating, vitamins, nutraceutical, chronic diseases

## Abstract

The unpredictable nature of new variants of coronavirus 2 (SARS-CoV-2)—highly transmissible and some with vaccine-resistance, have led to an increased need for feasible lifestyle modifications as complementary therapies. Systemic inflammation is the common hallmark of communicable diseases like severe coronavirus disease 2019 (COVID-19) and non-communicable chronic diseases (NCDs) such as obesity, cardiovascular diseases (CVD), diabetes mellitus, and cancers, all for which mitigation of severe outcomes is of paramount importance. Dietary quality is associated with NCDs, and intermittent fasting (IF) has been suggested as an effective approach for treatment and prevention of some NCDs, similar to that of caloric restriction. There is a paucity of high-quality data from randomized controlled trials regarding the impact of IF and the intake of specific nutrients on inflammation and post-infection outcomes in patients with severe acute respiratory syndrome coronavirus 2 (SARS-CoV-2). The current review of recent literature was performed to explore the immunomodulatory roles of IF regimens and supplements involving the intake of specific nutrients including vitamins (A, B, C, D, and E), zinc, and nutraceuticals (n-3 polyunsaturated fatty acids, quercetin, and probiotics) on inflammatory and oxidative stress markers, with consideration of how they may be related to SARS-CoV-2.

## 1. Introduction

A new wave of a subvariant of concern of the “omicron” variant of severe acute respiratory syndrome coronavirus 2 (SARS-CoV-2)—which poses very high risk of infection—is spreading worldwide, continuing the evolving pandemic that is now in its third year [1]. The efficacy of current vaccines against this heavily mutated variant is still unclear [2]. The unpredictable nature of new variants of SARS-CoV-2 and the poorly characterized and poorly understood post-acute sequelae (PASC) of coronavirus disease 2019 (COVID-19) have led to an increased need for feasible lifestyle modifications as complementary therapies to vaccines to reduce disease severity, particularly given that those effects wane over a period of months [3]. Identification of effective and feasible complementary therapies is critical to provide protection against the severity of both acute and PASC outcomes, since vaccinating a majority of the world population every 6 months or even more frequently is unlikely to be financially or logistically possible.

A key characteristic of severe presentations of acute SARS-CoV-2 infection involves overactive host inflammatory responses, with a substantial proportion of severe outcomes such as hospitalizations and deaths from COVID-19 linked to hyper-inflammation [4]. Inflammation and oxidative stress play pivotal roles in the progression of infectious diseases including COVID-19 [5]. Evidence suggests that high sensitivity (hs) C-reactive protein (CRP), interleukin (IL)-6, and matrix metalloproteinases (MMPs) are among the most important biomarkers of COVID-19 severity, similar to the chronic conditions involved in vascular aging [6,7,8,9]. Lactate dehydrogenase (LDH) and hsCRP are also biomarkers of respiratory failure in patients with COVID-19 [10]. Furthermore, it is well established that elevated levels of other inflammatory markers are common in COVID-19 patients. These markers include IL-1β, IL-7, IL-8, IL-18, interferon (IFN)-γ, tumor necrosis factor (TNF)-α, procalcitonin (PCT), serum ferritin, and erythrocyte sedimentation rate (ESR) [11,12].

Interestingly, hs-CRP, IL-6, and many of the other inflammatory biomarkers mentioned above, are also markers of risk in NCDs such as coronary artery disease and diabetes [13,14,15,16]. A wealth of evidence indicates that treatments that reduce inflammation, assessed using these biomarkers, also protect against major adverse cardiovascular events [17,18,19]. Some of these treatments may include simple nutritional therapies such as vitamin D supplementation in patients with vitamin D deficiency [20]. Many studies suggest that a high-quality diet, calorie restriction-induced weight loss (>5%), and intermittent fasting (IF), lower the risk of NCDs including diabetes and coronary artery disease [21,22,23,24,25,26,27,28,29,30,31,32], which may be due in part to reduction of chronic inflammation. If so, it may also be that IF and some simple specific nutrients may play a role in boosting immunity in response to SARS-CoV-2 infection, as has been hypothesized in previous reviews [33,34,35,36]. Yet, there is a paucity of comprehensive data from randomized controlled trials (RCTs) regarding the impact of IF and other simple nutritional therapies on inflammation generally, and specifically related to their effects on responses to SARS-CoV-2. While weight loss is known to reduce inflammation and to control risk factors related to both COVID-19 outcomes and NCDs, some dietary regimens, including IF, may impact inflammation and other pathways independently of weight loss [30,37,38]. The current review of recent literature was performed to explore the immunomodulatory roles of dietary supplements (including some vitamins, minerals, and nutraceuticals) and IF on inflammatory and oxidative stress markers that may be related to SARS-CoV-2 or host responses to infection, and also have some connection to reduction of chronic inflammation linked to NCDs.

## 2. Methodology of Literature Search

A literature search of the PubMed and Google Scholar databases was conducted to identify peer-reviewed articles published from January 2017 to March 2022 that involved assessments of markers of inflammation or oxidative stress. The search terms for dietary supplementation articles included the following: “vitamin”, “nutrient”, “mineral”, “antioxidant”, “nutraceuticals”, “polyphenols”, “resveratrol”, “quercetin”, “probiotic”, “omega-3”, combined with “infection”, “inflammation”, “inflammatory markers”, “immune response”, “immunity”, “oxidative stress”, “CVD”, “obese adults”, “obesity”, “overweight”, “‘SARS-CoV-2′”, and “COVID-19.” The search terms for intermittent fasting articles included the following: “intermittent fasting”, “alternate-day fasting”, “time-restricted eating”, “5:2 diet”, “Ramadan fasting”, combined with “infection”, “inflammation”, “inflammatory markers”, “immune response”, “immunity”, “oxidative stress”, “CVD”, “obese adults”, “obesity”, “overweight”, “SARS-CoV-2”, and “COVID-19.” Filters were set to allow only “human” studies, and studies written in the “English” language.

## 3. The Impact of Dietary Supplements on Inflammatory Markers and in Response to SARS-CoV-2

### 3.1. Vitamins and Minerals

Supplementation with vitamins such as A, B, C, D, and E is thought to play a significant role in the severity of COVID-19 infection by reducing inflammation, time to recovery, and preventing lung fibrosis [39,40]. To our knowledge, no RCTs have tested the effects of supplementation with vitamin A, B, or C alone, on COVID-19 severity; however, in a 7-day randomized placebo-controlled trial of 60 COVID-19 patients admitted to intensive care unit (ICU), those who received a combination of vitamins A (25,000 IU daily), D (600,000 IU; one dose), E (300 IU twice daily), C (500 mg four times daily), and a B-complex ampule (daily), demonstrated significant reductions in the duration of hospitalization, TNF-α, Il-6, erythrocyte sedimentation and hs-CRP, but not IFN-γ, as compared to a placebo [41]. In contrast, 10-days of standard treatment plus high doses of vitamin C (2 g), Melatonin (6 mg), and Zinc (50 mg), was not effective for lowering inflammatory markers or length of hospitalization in 20 patients with severe COVID-19 [42].

Accumulating data highlight the immunomodulatory role of vitamin D, and link hypovitaminosis D (25 OHD ≤ 20 ng/mL) with hyperinflammation (i.e., the so-called “cytokine storm”) and elevated risk of mortality in patients with COVID-19 [43,44,45,46]. Therefore, supplementation with vitamin D is suggested to attenuate the risk of the cytokine storm, and the severity of COVID-19 [43,47]. The impact of vitamin D on inflammatory markers and in response to SARS-CoV-2 infection has been explored in several RCTs (see Table 1). The current state of evidence from RCTs indicates that higher doses of daily intake of vitamin D can reduce the levels of inflammatory markers like IL-6, hsCRP, and time to recovery in COVID-19 patients [48,49]. Ten days of supplementation with 60,000 IU/day of vitamin D in combination with standard treatment significantly reduced IL-6, hsCRP, LDH, ferritin, and Neutrophil/Lymphocyte ratio, in 87 hospitalized COVID-19 patients with vitamin D deficiency (D < 30 ng/m) as compared with the controls, who received standard treatment for 8 to 10 days [49]. Similarly, in a 2-week trial, oral intakes of two different doses of vitamin D3 (5000 IU vs. 1000 IU) resulted in significant decreases in plasma IL-6 versus baseline, with no between group differences, while hsCRP levels remained unchanged in both groups [48]. Furthermore, time required for resolving cough symptoms with D3 supplementation (5000 IU) was significantly shorter compared to the comparison group [48]. Rastogi et al. investigated the effects of high-dose vitamin D supplementation (60,000 IU) as compared with control in 40 SARS-CoV-2 RNA positive individuals. Patients with vitamin D deficiency (25(OH) D < 20 ng/mL) who were positive for SARS-CoV-2 RNA, with mild or no symptoms, significantly improved with regard to viral SARS-CoV-2 RNA clearance (62.5% vs. 20.8%) and fibrinogen levels [50]. In contrast, a single dose of vitamin D was ineffective for lowering inflammatory markers and for the treatment of patients with severe COVID-19 [51,52,53].

Evidence for the potential role of zinc supplementation for COVID-19 infection is inconclusive [54,55,56]; with only one 28-day RCT of 191 patients with COVID-19 having investigated the effects of zinc supplementation (50 mg of zinc twice daily) combined with chloroquine/hydroxychloroquine (CQ/HCQ) on inflammatory markers. The study results indicated no significant changes in hs-CRP levels or clinical recovery time [57].

**Table 1:** Summary of Randomized controlled trials (RCTs) on the impact of dietary supplements on inflammatory markers and in response to SARS-CoV-2.

Authors/Year/Country	Duration	Participants	Study Design	TNF-α	IL-1β	IL-4	IL-6	IL-10	CRP	IFN-γ	Other Outcomes
Rastogi et al., 2020 [50] India	7-d	40 SARS-CoV-2 RNA positive individuals	RCT: 1.Daily oral cholecalciferol (60,000 IU) with therapeutic target 25(OH)D > 50 ng/mL 2. Control	-	-	-	-	-	Ø	-	↓ † Fibrinogen ↑ † Negative conversion of SARS-CoV-2 RNA (62.5% vs. 20.8%)
Murai et al., 2021 [53] Brazil	-	237 patients hospitalized for moderate to severe COVID-19	RCT: 1.A single oral dose of 200,000 IU of vit. D3 2. Placebo	-	-	-	-	-	Ø	-	Ø Same LOS (median of 7.0 vs. 7.0 days)
Lakkireddy et al., 2021 [49] India	8–10-d	87 Patients hospitalized for COVID-19 Vit. D < 30 ng/m	RCT: 1. 60,000 IU/day of oral vitamin D +standard treatment 2. Only standard treatment	-	-	-	1. ↓ † 2. Ø		1. ↓ *† 2. ↓ *	-	↓ *† LDH, ferritin, N/L ratio
Beigmohammadi et al., 2021 [41] Iran	7-d	60 ICU-admitted COVID-19 patients	RCT: 1. Oral Vit. A (25,000 IU) daily, vit.D (600,000 IU; one dose), vit. E (300 IU twice daily), vit. C (500 mg four times daily), and one amp daily of B complex 2. Placebo	1. ↓ *† 2. ↓ *	-	-	1. ↓ *† 2. ↓ *	-	1. ↓ *† 2. ↓ *	1. ↓ * 2. ↓ *	↓ † Hospitalization rate and ESR in treatment group
Sabico et al., 2021 [48] Saudi Arabia	14-d	69 patients COVID-19 and sub-optimal vit. D status	RCT: 1.Oral vit. D3 (5000 IU) 2.Oral vit. D3 (1000 IU)	-	-	-	1. ↓ * 2. ↓ *	-	1. Ø 2. Ø	-	↓ † Time to recovery in resolving cough with D3 (5000 IU) vs. D3 (1000 IU)
Abd-Elsalam et al., 2021 [57] Egypt	4 weeks	191 patients with COVID-19	RCT: 1.CQ/HCQ + 220 mg of zinc sulfate twice daily 2. HCQ only	-	-	-	-	-	Ø	-	Ø Clinical efficacy of HCQ
Di Pierro et al., 2021 [58] Italy	2 weeks	42 COVID-19 outpatients	RCT: 1. Quercetin (500 mg/day (first week) and of 1000 mg/day (second week) 2. Standard of care	-	-	-	-	-	Ø	-	↓ † LOS, virus clearance, symptoms frequency, LDH, ferritin
Doae et al., 2021 [59] Iran	2 weeks	128 critically ill COVID-19 patients	RCT: 1. 1000 mg omega-3 daily (400 mg EPAs and 200 mg DHAs) 2. Control	-	-	-	-	-	-	-	↑ † 1-month survival rate, pH, HCO_3_, and Be ↓ † Levels of BUN, Cr, and K in the treatment group
Darban et al., 2021 [42] Iran	10-d	20 patients with severe COVID-19	Pilot RCT: 1. Standard care + oral zinc sulfate (220 mg containing 50 mg zinc) , oral melatonin (6 mg, q6hr), and intravenous vit. C (2 g) 2. Standard care alone	-	-	-	-	-	1. ↓ * 2. ↓ *	-	Ø LOS
Sedighian et al., 2021 [60] Iran	2 weeks	30 patients with COVID-19	Single blind RCT: 1. Hydroxychloroquine + 2 g DHAs and EPAs 2. Hydroxychloroquine	-	-	-	-	-	1. ↓ †	-	† Body pain and fatigue in the treatment group Ø Olfactory
Cannata-Andía et al., 2022 [52] Spain	-	543 patients with moderate to severe COVID-19	RCT: 1. A single-oral bolus of 100,000 IU of cholecalciferol 2. Control	-	-	-	Ø	-	Ø	-	Ø Hospitalization rate and death
Shohan et al., 2022 [61] Iran	7-d	60 patients with severe COVID-19	RCT: 1. Quercetin (1000 mg daily) + antiviral drugs 2. Antiviral drugs	↓ *	↓ *	-	↓ †	-	↓ †	-	↓ † ALP, LDH Ø Mortality, duration of ICU-admission
Pimentel et al., 2022 [62] Brazil	7-d	43 adult patients with COVID-19	RCT: 1. Two 200 mL units of high-protein nutritional supplement (arginine, omega-3 fatty acids and nucleotides) over 24 h 2. Two 200 mL units of high-protein nutritional supplement alone	-	-	-	-	-	1. ↓ † 2. Ø	-	↑ Lymphocytes in the treatment group ↓ Lymphocytes in the control group
Fernandes et al., 2022 [51] Brazil	-	200 patients with moderate to severe COVID-19	RCT: 1. Single oral dose of vit. D_3_ (200 000 IU) 2. Placebo	Ø	Ø	Ø	Ø	Ø	-	Ø	-
Gutiérrez-Castrellón et al., 2022 [63] Spain	30-d	293 COVID-19 outpatients	RCT: 1. Probiotic (Lactiplantibacillus plantarum stains KABP022, KABP023 and KABP033 = Pediococcus acidilactici strain KABP021) 2. Placebo	-	-	-	-	-	1. ↓ † hs-CRP Only on day 15	-	↓ † Complete remission (53.1% in probiotic group vs. 28.1% in placebo; *p* < 0.001)

-: Not measured. Ø: Non-significant difference between groups. * *p* < 0.05, Significantly different from baseline (within group effect). † *p* < 0.05, Significantly different from the control or. comparison group. TNF-α: tumor necrosis factor -α; IL-1β: Interleukin-1β; IL-4: Interleukin-4; IL-6:Interleukin-6; IL-10: Interleukin-10; IFN-γ: Interferon gamma; CQ/HCQ:Chloroquine/hydroxychloroquine; HCQ: Hydroxychloroquine; ALP: Alkaline phosphatase; Lactate dehydrogenase; Be: Base excess; BUN: Blood urea nitrogen; Cr: creatinine; LOS: Length of hospital stay; LDH: Lactate dehydrogenase; N/L ratio: Neutrophil/Lymphocyte ratio; ESR: Erythrocyte sedimentation rate; DHA: Docosahexaenoic acid; EPA: eicosapentaenoic acid.

### 3.2. n-3 Polyunsaturated Fatty Acids (PUFAs)

It is well known that n-3 PUFAs including eicosatetraenoic acid (EPA) and docosahexaenoic acid (DHA) have anti-inflammatory properties [64,65,66]. The current state of evidence from RCTs suggests that supplementation with n-3 fatty acids appears to be effective for alleviating clinical symptoms and inflammatory responses in patients with COVID-19 (see Table 1). Three of the trials included in this review investigated the efficacy of n-3 PUFAs for COVID-19 outcomes [59,60,62]. Supplementation with daily omega-3 capsules (1000 mg; 400 mg EPAs and 200 mg DHAs) for 2 weeks resulted in significantly higher 1-month survival rates in 128 ICU patients with COVID-19 when compared to the control patients [59]. The study also reported meaningful improvements in respiratory and renal function parameters such as arterial PH, bicarbonate, and other base excess in the treatment group. Similarly, DHA and EPA supplementation (2 g) plus hydroxychloroquine, significantly reduced hs-CRP, body pain, and fatigue compared to hydroxychloroquine alone in 30 patients with COVID-19 [60]. In agreement with those results, a 7-day trial in 43 patients with COVID-19 who were treated with oral immuno-nutrient supplements containing arginine, omega-3 fatty acids, and nucleotides (Two 200 mL units over 24 h), indicated meaningful reductions in hs-CRP when compared with the patients who were given only high-protein nutritional supplements (Two 200 mL units) [62].

### 3.3. Quercetin

Quercetin—a polyphenol with antioxidant and anti-inflammatory properties—is widely known to have favorable effects on inflammation and infection [67,68,69]. Di Pierro et al. reported that in 42 outpatients with COVID-19, two weeks of quercetin therapy (500–1000 mg daily) significantly diminished the rate (−68.2%) and length (−76.8%) of hospitalization, the need for non-invasive oxygen therapy (−93.3%), and the mortality rate (although events were very limited: none vs. 3 people) compared to the control (standard of care) [58]. Furthermore, significant reductions in LDH and ferritin levels were reported in the treatment group vs. control, while hs-CRP remained unchanged [58]. Unlike the 2-week trial by Di Pierro et al., a shorter 7-day RCT including 60 patients with severe COVID-19, treated with daily quercetin (1000 mg) along with antiviral drugs, compared with control (only antiviral drugs), did not alter mortality or ICU-admission duration significantly. Notably, significant reductions in inflammatory markers such as TNF-α, IL-1β, IL-6, hs-CRP, ALP, and LDH were shown in the treatment group as compared to the control or baseline [61].

### 3.4. Probiotics

High quality evidence from systematic reviews and meta-analyses of RCTs, indicates that probiotics may have a favorable role for responses to infections [70]. In a recent one-month RCT of 293 outpatients with COVID-19, supplementation with four-strain probiotics consisting of Lactiplantibacillus plantarum KABP033 (CECT30292), L. plantarum KABP022 (CECT7484), L. plantarum KABP023 (CECT7485), and Pediococcus acidilactici KABP021 (CECT7483), produced significant reductions in remission rates vs. placebo (53.1% in probiotic group vs. 28.1% in placebo [63].

## 4. The Potential Impact of IF on Inflammatory Markers

To date, no RCTs have investigated the effects of IF on SARS-CoV-2 infection or severity of COVID-19 outcomes, although epidemiologic evidence regarding periodic fasting suggests that fasting in some forms may prevent severe COVID-19 outcomes such as hospitalization and death [71], perhaps in part, through the control of hyper-inflammation. The impacts of IF on markers of inflammation and oxidative stress are summarized below:

### 4.1. Interluekins

Current evidence from RCTs reveals that time-restricted eating (TRE) may be effective for reducing interleukins IL-1β, IL-6 and IL-8 (see Table 2.) Of the 11 TRE trials included in this review, only one trial assessed IL-1β levels. This 12-month trial of 20 healthy athletes reported significant reductions in both IL-1β and IL-6 levels in the TRE group (3 meals; 1 p.m., 4 p.m., and 8 p.m.) compared with normal diet control group (3 meals; 8 a.m., 1 p.m., and 8 p.m.) [72]. However, IL-6 levels have remained unchanged in other TRE studies [30,32,73,74]. Xie et al. conducted a 5-week trial examining plasma IL-8 levels in 82 healthy participants who had normal weight, when following early TRE (6 a.m.–3 p.m.), mid-day TRE (11 a.m.–8 p.m.), and as compared with an ad libitum intake control group, and showed a significant reduction in IL-8 levels vs. control when following early TRE [75]. Similarly, results of observational and clinical trials from Ramadan IF (fasting from sunrise to sunset) studies suggest that this form of IF may be effective for counterbalancing inflammatory interleukins such as IL-1β, IL-2, Il-6, IL-8, and IL-10 [76,77,78,79,80,81,82,83,84,85] (see Table 3). In a one-month RCT in 28 men with obesity conducted by Zouhal et al., Ramadan IF produced meaningful reductions in plasma IL-6 levels vs. control. Unlike the results from TRE and Ramadan IF trials, other popular types of IF, including alternate-day fasting (ADF) and twice-weekly 24-h fasting (commonly called “5:2 diets”) have not shown effects on inflammatory interleukins including IL-1β, IL-6, and IL-10 [28,86,87,88,89,90,91,92] (see Table 4). The effects of 24-h prolonged fasting on inflammatory markers has been tested in two single-arm trials by Han et al. with results indicating significant improvements in IL-1β, IL-2, IL-4, IL-5, IL-13, IL-17 and IL-22 as compared to postprandial responses measured 3 h after refeeding with isocaloric breakfast meal (500 kcal) [93,94].

### 4.2. TNF-α

Studies of the impact of different IF regimens on TNF-α have reported inconsistent results (see Table 2, Table 3 and Table 4). Early TRE and Ramadan IF have been shown to significantly improve TNF-α levels vs. control and/or baseline, while mid-day TRE has not shown such improvements in two out of the three TRE trials included in this review [32,72]. Likewise, ADF appears to be ineffective for reducing plasma TNF-α, as two ADF trials in adults with overweight or obesity and metabolic syndrome, which tested changes in TNF-α have not demonstrated significant improvements following ADF regimens [86,91]. Of the two twice-weekly fasting studies which assessed TNF-α, one RCT in 43 adults with central obesity showed significant decreases (−18%) vs. baseline, but no between group differences when compared with a caloric restriction arm [90].

### 4.3. C-Reactive Protein

There has also been inconsistency in the results of various trials of IF regimens on hs-CRP levels, with most studies indicating no effects (see Table 2, Table 3 and Table 4). Of the seven TRE studies included in this review, only two trials (4 to 5 week durations) reported significant improvements in hs-CRP levels (−45% and −42%) in overweight and obese participants vs. a control or as compared with baseline [96,97]. In two TRE studies, only hs-CRP was examined and was unchanged [95,98]. Hs-CRP levels were not altered in short-term (8 weeks) ADF studies, whereas longer duration trials (17 and 24 weeks; with a high protein diet) have significantly decreased hs-CRP (−48% and −19%) versus CR or baseline, respectively [87,89,91]. In addition, twice-weekly fasting did not appear to affect hs-CRP levels [28,38,88,90]. Furthermore, only one observational Ramadan IF study in 83 patients with non-alcoholic fatty liver disease, has shown significant decreases in hs-CRP levels vs. control and baseline following Ramadan IF after 30 days [76].

### 4.4. IGF-1

IGF-1 levels remain unchanged with most IF trials that recruited participants with overweight and obesity. In athletes, however, two RCTs conducted by Moro et al. reported significant reductions in IGF-1 levels with TRE as compared to control and baseline after both 4 weeks and 12 months [72,74]. IGF-1 significantly increased (34%) in a single arm trial of early TRE (8 a.m.–4 p.m.) in 15 overweight and obese women with Polycystic ovary syndrome [97]. There is lack of consistency in IGF-1 results of observational Ramadan IF studies (see Table 3).

### 4.5. Interferon Gamma and Other Inflammatory Markers

Interferon gamma (IFN-γ), a proinflammatory cytokine that plays a significant role in immune responses, has been investigated in only two 24-h prolonged fasting studies by Han and colleagues. These studies indicate that IFN-γ levels were significantly reduced when compared with 3 h after refeeding with isocaloric breakfast meal (500 kcal) in a total of adults who fasted for 24 h [93,94]. Matrix metalloproteinase 9 (MMP-9) was assessed in one observational Ramadan IF study in 34 healthy adults [85]. The levels MMP-9 were determined at five different time periods within 27–29 days of fasting during the month of Ramadan. While MMP-9 levels remained unchanged from mid-month (days 14–16) to one week after Ramadan, they were significantly lower vs. baseline at the one month after Ramadan timepoint (see Table 3). Changes in CD40 ligand have also been tested in two studies, with only one 8-week RCT of twice-per-week fasting in 39 adults with metabolic syndrome, demonstrating a meaningful reduction in CD40 ligand vs. control after 8 weeks [92].

### 4.6. Oxidative Stress

8-Isoprostane is a marker of oxidative stress that is associated with COVID-19 [99]. To date, only two trials have examined 8-Isoprostane levels following TRE [30,32], with significant decreases in 8-Isoprostane reported in both studies compared to both control and baseline. Early TRE (eTRE; 6-hr eating window, last meal before 3 pm) showed a 14% reduction in 8-Isoprostane in the eTRE arm versus control (12-hr. eating window) in 12 prediabetic men after 5 weeks. In addition, restricting eating windows to four (3–7 p.m.) and six hours (1–7 p.m.) significantly reduced 8-Isoprostane levels in both groups (37% and 34%, respectively) vs. control (ad libitum intake) in adults with obesity following 8 weeks [32]. In contrast, short-term (6 weeks) eucaloric TRE with self-selected eating windows did not significantly enhance plasma oxidized LDL or the oxidized total LDL ratio in non-obese healthy older adults [73].

## 5. Conclusions and Future Directions

Evidence suggests that intermittent fasting and supplementation with vitamin D, and nutraceuticals including quercetin, n-3 fatty acids, and probiotics, impact inflammation in canonical pathways related to both NCDs and infectious diseases including COVID-19 and, thus, should reduce COVID-19 severity. A lack of high-quality evidence exists for the support of such efficacy of supplementation with vitamins A, B, and C, as well as zinc in COVID-19 patients. Further investigation of these fasting and supplementation effects is indicated. Observational studies report that some diets (e.g., the Mediterranean diet, plant-based diets) may reduce SARS-CoV-2 severity, potentially—at least in part—because the foods contain antioxidants and other components with anti-inflammatory properties [100,101,102,103], but further research is needed on such diets.

Ultimately, the impact of intermittent energy restriction methods on inflammation during energy restriction may be acute but transient, contrasting with longer-term persistent changes to basal levels of inflammation. Future randomized controlled trials are needed to elucidate the effects of intermittent fasting on NCDs and on inflammatory host responses to infection.

## Figures and Tables

**Table 2:** Summary of clinical trials on the impact of time-restricted eating (TRE) on inflammatory markers and oxidative stress.

Authors/Year/ Country	Duration	Participants	Study Design	8-Isoprostane	TNF-α	IL-1β	IL-6	IL-8	CRP	IGF-1
Sutton et al., 2018 [30] US	5 weeks	12 prediabetic men	RCT crossover: 1. Eucaloric eTRE (6-hr eating window, last meal before 3 pm) without weight loss 2. Control (12-hr. eating window)	1. ↓14%† 2. Ø	-	-	Ø	-	Ø ^hs-CRP^	-
Jamshed et al., 2019 [37] US	4-day	11 overweight adults	RCT crossover: 1. eTRE (8 a.m.–2 p.m.) 2. Control (8 a.m.–8 p.m)	-	-	-	-	-	-	Ø
Martens et al., 2020 [73] US	6 weeks	22 healthy non-obese older adults	RCT: 1. Eucaloric TRE (16/8)- without weight loss 2. Control	-	-	-	Ø	-	Ø	-
Wilkinson et al., 2020 [95] US	12 weeks	19 adults with MetSyn	Single arm: 10-h TRE (self-selected eating window)	-	-	-	-	-	Ø ^hs-CRP^	-
Cienfuegos et al., 2020 [32] US	8 weeks	49 obese adults	RCT: 1. 4-h TRE (3–7 p.m.) 2. 6-h TRE (1–7 p.m.) 3. Control	1. ↓37% *^,†^ 2. ↓34% *^,†^ 3. Ø	Ø	-	Ø	-	-	-
McAllister et al., 2020 [96] US	4 weeks	22 men BMI: 28.5 ± 8.3	RCT: 16:8 TRE 1. Isocaloric TRE 2. Ad libitum TRE	-	-	-	-	-	1. ↓45† ^hs-CRP^ 2. ↓14	-
Moro et al., 2020 [74] Italy	4 weeks	16 elite under -23 cyclists	RCT: 1. TRE (10 a.m.–6 p.m.) 2. Control (7 a.m.–9 p.m.)	-	Ø	-	Ø	-	-	1. ↓14% * 2. Ø
Moro et al., 2021 [72] Italy	12 months	20 healthy resistance-trained males	RCT: 1. mTRE (3 meals;1 p.m.,4 p.m. and 8 p.m.) 2. Control (8 a.m., 1 p.m., and 8 p.m.)	-	1. ↓ *† 2. Ø	1. ↓ *† 2. Ø	1. ↓ *† 2. Ø	-		1. ↓ *† 2. ↑ †
Li et al., 2021 [97] China	5 weeks	15 overweight and obese women with PCOS	Single arm: eTRE (8 am–4 pm) 1-week baseline weight stabilization + 5-week trial	-	-	-	-	-	↓42% * ^hs-CRP^	↑34% *
Kotarsky et al., 2021 [98] US	8 weeks	21 overweight and obese adults	RCT: 1. mTRE (12–8 p.m.) + concurrent exercise training 2. Normal eating + concurrent exercise training	-	-	-	-	-	Ø ^hs-CRP^	-
Xie et al., 2022 [75] China	5 weeks	82 healthy adults	RCT: 1. e-TRE (6 a.m.–3 p.m.) 2. mTRE (11 a.m.–8 p.m.) 3. Control (ad lib intake)	-	1. † 2.Ø 3. Ø	-	-	1. † 2.Ø 3. Ø	Ø	-

-: Not measured. Ø: Non-significant difference between groups. * *p* < 0.05, Significantly different from baseline (within group effect). † *p* < 0.05, Significantly different from the control or comparison group; TNF-α: tumor necrosis factor -α; IL-1β: Interleukin-1β; IL-6: Interleukin-6; IL-8: Interleukin-8; CRP: C-reactive protein; IGF-1: Insulin-like growth factor *1*TRE: Time-restricted eating; eTRE: Early TRE; mTRE: Mid-day TRE; MetSyn: Metabolic syndrome; PCOS: Polycystic ovary syndrome.

**Table 3:** Summary of studies on the impact of Ramadan fasting on inflammatory markers.

Authors/Year/ Country	Duration	Participants	Study Design	MMP-9	TNF-α	IL-1β	IL-2	IL-6	IL-8	IL-10	CRP	IGF-1
Aliasghari et al., 2017 [76] Iran	30-d	83 patients with NAFLD	Observational Ramadan fasting 1.Ramadan fasting (n = 42) 2.Control (n = 41)	-	-	-	-	1. ↓ *† 2. ↓ *	-	-	1. ↓ *† ^Hs-CRP^ 2. ↓ *	-
Mohammad zade et al., 2017 [77] Iran	30-d	23 healthy men	Observational Ramadan fasting	-	-	-	-	Ø	-	-	Ø	-
Mushtaq et al., 2019 [78] Pakistan	29-d	110 normal, overweight, and obese men (n = 55) and women (n = 55)	Observational Ramadan fasting (1st vs. 29th day of Ramadan just before Iftar) + dietary recommendation (oily foods prohibited at Iftar (breaking of fast time), plus white oats provided (bran diet) for Sahar (onset of fasting time) meal	-	↓16% * Ψ men ↓11% * Ψ women	-	-	-	-	-	-	-
Almeneessier et al., 2019 [79] Saudi Arabia	14-d	12 healthy men	Single arm Ramadan fasting (from dawn to sunset)	-	-	↓ *	-	↓ *	↓ *	-	-	-
Rahbar et al., 2019 [80] Saudi Arabia	30-d	34 healthy men	Observational Ramadan fasting	-	-	-	↓ *	-	-	-	---	↓ *
Faris et al., 2019 [81] UAE	30-d	57 overweight and obese adults	Observational Ramadan fasting	-	↓ *	-	-	↓ *	-	↑*	-	↓ *
Mansoor et al., 2020 [81] Iraq	21-d	20 healthy young women aged 19–20 y	Observational Ramadan fasting	-	↓ *	-	-	↓ *	-		-	-
Lubis and Pase. 2020 [83] Indonesia	30-d	30 obese adults	Observational Ramadan fasting	-	-	-	-	↓ *	-	-	-	-
Zouhal et al., 2020 [84] Tunisia	30-d	28 obese men	RCT: 1. Ramdan fasting 2. Control	-	1. ↓ † 2. ↓ *	-	-	1. ↓ † 2. Ø	-	-	1. Ø 2.Ø	-
Riat et al., 2021 [85] Germany	27–29-d	34 healthy adults	Observational Ramadan fasting (17–18 h) T1: baseline: 6–8 d before Ramadan T2: Mid of Ramadan (day 14–16) T3: End of Ramadan (day 27–29) T4: One week after Ramadan T5: One month after Ramadan	T2: Ø T3: Ø T4: Ø T5: ↓ *	-	-	-	-	T2: ↑* T3: ↓¥ T4: ↓¥ T5: ↓¥	-	-	T2: ↑* T3: ↓¥ T4: Ø T5: ↑*

-: Not measured. ∅: Non-significant change between groups. * *p* < 0.05, Significantly different from baseline (within group effect). † *p* < 0.05, Significantly different from the control or comparison group. ¥ *p* < 0.05, Significantly different from T2. MMP-9: *Matrix metalloproteinase;* TNF-α: tumor necrosis factor -α; IL-1β: Interleukin-1β; IL-2: Interleukin-2; IL-6: Interleukin-6; IL-8: Interleukin-8; IL-10: Interleukin-10; CRP: C-reactive protein; IGF-1: Insulin-like growth factor 1; NAFLD: Non-alcoholic fatty liver disease. Ψ: Significant reduction in only obese group (30 men, 30 women).

**Table 4:** Summary of Randomized controlled trials (RCTs) on the impact of intermittent calorie restriction on inflammatory markers.

Authors/ Year/ Country	Duration	Participants	Study Design	TNF-α	IL-1β	IL-2	IL-4	IL-5	IL-6	IL-13	IL17	IL-22	CRP	IFN-γ	CD40 Ligand	IGF-1
Trepanowski et al., 2018 [86] US	24 weeks	69 overweight and obese	RCT: 1. ADF (25% energy needs on fast days;125% on fed days) 2. CR (75% energy needs daily) 3. Control (100% energy needs daily)	1. Ø 2. Ø 3. Ø	-	-	-	-	1. Ø 2. Ø 3. Ø	-	-	-	-	-	-	-
Bowen et al., 2018 [87] Australia	24 weeks	162 overweight and obese adults	RCT: 1. High protein, ADF + CR (1 Ad lib intake d/wk) 2. CR	-	-	-	-	-	-	-	-	-	1. ↓19* ^hs-CRP^ 2. Ø	-	-	-
Schübel et al., 2018 [28] Germany	12 weeks	144 overweight and obese adults	RCT: 12-week trial 5:2 vs. CR and control +38 wk (12 wk maintenance + 26 wk follow up)	-	-	-	-	-	-	-	-	-	Ø	-	-	Ø
Sundfor et al., [88] 2018 Norway	52 weeks	112 obese adults	RCT: 5:2 diet vs. CR + Med diet	-	-	-	-	-	-	-	-	-	Ø ^at 6 months^	-	-	-
Cho et al., 2019 [89] South Korea	8 weeks	31 overweight and obese adults	RCT: ADF vs. usual diet with or without exercise	-	-	-	-	-	-	-	-	-	Ø ^hs-CRP^	-	-	-
Pinto et al., 2019 [90] UK	4 weeks	43 adults with central obesity	RCT: 1. 5:2 diet 2. CR	1. ↓18% * 2.Ø	1. Ø 2. Ø	-	-	-	1. Ø 2. Ø	-	-	-	-	-	-	-
Razavi et al., 2021 [91] Iran	17 weeks	75 adults with MetSyn	RCT: 1. ADF (400–600 kcal on fast days) 2. CR (75% energy restriction daily)	1. Ø 2. Ø	-	-	-	-	1. Ø 2. Ø	-	-	-	1. ↓48% * † ^hs-CRP^ 2. ↓26% *	-	-	-
Han et al., 2021 [94] US	1-day	20 adults	Single arm: 24-h prolonged fasting vs. post-prandial response, 3 h after isocaloric breakfast (500 kcal)	-	↓ *	↓ *	↓ *	↓ *	-	-	↓ *	-	-	↓ *	-	-
Han et al., 2021 [93] US	1-day	21 adults	Single arm: 24-h prolonged fasting vs. post-prandial response, 3 h after isocaloric breakfast (500 kcal)	-	-	-	Ø	↓ *	-	↓ *	↓ *	↓ *	-	↓ *	-	-
Bartholomew et al., 2021 [38] US	26 weeks	103 adults; ≥1 MetSyn component or T2D	RCT: 1. 5:2 diet for 4 weeks; followed by fasting once a week for 22 weeks 2. Ad libitum control	-	-	-	-	-	-	-	-	-	Ø	-	-	-
Guo et al., 2021 [92] China	8 weeks	39 adults with MetSyn	RCT: 1. 5:2 diet (two days fasting per week) 2. Control	Ø	-	-	-	-	Ø	-	-	-	-	-	1. ↓ † 2. Ø	-

-: Not measured. Ø: Non-significant change between groups. * *p* < 0.05, Significantly different from baseline (within group effect). † *p* < 0.05, Significantly different from the control or. comparison group. TNF-α: tumor necrosis factor -α; IL-1β: Interleukin-1β; IL-2: Interleukin-2 IL-4: Interleukin-4 IL-5: Interleukin-5; IL-6: Interleukin-6; IL-13: Interleukin-13; IL-17: Interleukin-17; IL-22: Interleukin-22; CRP: C-reactive protein; IFN-γ: Interferon gamma; IGF-1: Insulin-like growth factor 1; ADF: Alternate-day fasting. CR: Caloric restriction; MetSyn: Metabolic syndrome; Med diet: Mediterranean diet; T2D: Type 2 diabetes.

## Data Availability

Not applicable.
